# Retained Foreign Object Signals a Dangerous Atmosphere in the Operating Room

**DOI:** 10.7759/cureus.80301

**Published:** 2025-03-09

**Authors:** Daniele Rigamonti, Karen H Rigamonti, Alessandra S Rigamonti

**Affiliations:** 1 Neurosurgery,, Johns Hopkins Medicine, Pikesville, USA; 2 Anesthesiology, KHDR Consulting, Baltimore, USA; 3 Neurological Surgery, Brown University, Providence, USA

**Keywords:** harm, never event, retained foreign object, retained surgical item, rfo, rsi, se, sentinel event, unintentionally retained foreign object, urfo

## Abstract

A retained foreign object (RFO), also known as an unintentionally retained foreign object (URFO) or a retained surgical item (RSI), is an object retained after skin closure following an invasive procedure. After falls, it is the second most reported sentinel event (SE). Several factors increase the risk of RFO: intraoperative blood loss, longer duration of operation, more sub-procedures, lack of (or incorrect) surgical counts, more than one surgical team, and unexpected intraoperative factors. Unclear policies regarding the counting responsibility, the handling of surgical specimens, the involvement of two surgical teams, and the improper hand-off with a shift of the surgical technician represent other important contributing factors. Technologies, such as bar-coded sponges to aid in accurate counting and radiofrequency identification (RFID)-tagged sponges to provide intraoperative counting and detection, have shown to decrease the incidence of RFOs. However, the adoption of these technologies has been limited. Furthermore, the extremely high percentage of falsely “correct counts” points to the critical role of an unsafe operating ooom (OR) culture in the genesis of RFOs. We argue that the RFO is the dead canary: just as the dead bird signals the presence of lethal gas in the coal mine, the RFO signals the presence of a dangerous culture in the OR.

Eliminating RFOs requires a multipronged strategy. OR staff should be reminded that failure can and will happen and they need to remain vigilant. Every team member should be capable and responsible to prevent the compounding of errors. Counting policy should be standardized mandating when, what, how, and by whom surgical counts are performed and documented. When policy is ignored or violated, OR staff should be involved in reviewing and revising the policy. However, commitment to safety requires leadership to provide appropriate resources and to role model core organizational values.

## Introduction and background

It is well-documented that delivering care can, unfortunately, produce harm. Almost seventy years ago, in 1954, Beecher reported that anesthesia was more deadly than poliomyelitis and of sufficient magnitude to constitute a public health problem [1]. Sadly, between 4% and 10% of patients have complications associated with their medical treatment [2,3]. A study in 1999 estimated the number of people dying in any given year from medical errors occurring in hospitals to be 98,000 [4]. By 2016, that estimated number had risen to 250,000, exceeding those that die from motor vehicle accidents, breast cancer, or AIDS, three causes receiving far more public attention [5]. These reports led healthcare organizations to study the processes used by high-reliability organizations (HROs), like nuclear plants and air carriers, to eliminate errors leading to accidents. To reduce harm, healthcare organizations adopted similar processes and developed a new framework for analyzing risk and a culture of error recognition and accountability. Despite these efforts, healthcare is still not safe and harm continues to occur [6].

Any unexpected patient safety incident resulting in death, severe or permanent harm, or the risk of such, is called a “sentinel event”. A retained foreign object (RFO), also known as an unintentionally retained foreign object (URFO), or a retained surgical item (RSI), is an object (usually a gauze, a pad, or a surgical tool) that is retained in a body cavity after skin closure has occurred following an invasive procedure. An RFO is a preventable serious patient safety event that has been classified as a “never event” requiring mandatory reporting in some countries. After falls, the RFO is the second most common sentinel event (SE) reported to The Joint Commission (TJC) [7].

Its reported annual incidence ranges between 1/1,000 and 1/18,000 surgeries depending on the study [8,9]. Patients with RFO have significantly increased risks for pulmonary infection, wound infection, sepsis, longer length of stay, and higher costs [9,10]. For surgeons and hospitals, RFOs have considerable negative psychological and reputational impact. Furthermore, the economic and legal implications are significant. It is calculated that in the US, an RFO event generates additional healthcare costs averaging $70,000/case. This does not include liability settlements related to RFO, estimated at $150,000 per patient [10]. While it is disheartening to discover that healthcare is still not safe, it is even more surprising to discover that eminently preventable adverse events, like RFOs, continue to happen. We reviewed the published literature and our own experience to find a possible overlooked explanation for this persistent failure and to consider more effective remedies. 

## Review

In 1882, Doran mentioned a case of RFO leading to the death of the patient due to peritonitis [11]. In 1884, Dr. Wilson, a gynecological surgeon, published the first set of recommendations to avoid the occurrence of RFO. He listed counting of sponges/instruments used in the operation, making a record of it, and performing a tally at the closure of the operation. He recommended that the surgeon restrict the use of assistants, the number of instruments, and the number of sponges and reinforce collaboration within the OR team. He also insisted that the surgeon should do his own sponging, should use as few sponges as possible, preferably have a fixed number in use, should never allow a sponge to be divided during an operation, and should certify the count at the end [12]. In 1901, Schachner discussing a series of 155 RFO events, recognized that the critically important counting responsibility needs to be clearly assigned to two nurses, aids should be used in localizing the pads or the instruments, the use of small sponges should be discouraged, and as few as possible utensils should be used. He recognized that distraction and disruption of a routine (such as when the surgeon is facing an intraoperative disaster due to overwhelming hemorrhage or is forced to make significant changes in the intraoperative plan) represent important risk factors. He concluded that watchfulness has a critical role [13]. Gawande recognized that emergencies, unplanned changes in procedure, and higher body-mass index significantly increase the risk of retention of a foreign body after surgery [14].

A meta-analysis based on three early retrospective case-control studies (published between 2003 and 2013) reports that seven risk factors synergistically increase RFO risk. These include intraoperative blood loss >500 mL (odds ratio=1.6), duration of operation (odds ratio=1.7), >1 sub-procedure (odds ratio=2.1), lack of surgical counts (odds ratio=2.5), >1 surgical team (odds ratio=3.0), unexpected intraoperative factors (odds ratio=3.4), and incorrect surgical count (odds ratio=6.1). Changes in nursing staff, emergency surgery, body-mass index, and operation “after-hours” were not significantly associated with increased RSI/RFO risk [15]. Other factors contributing to the occurrence of RFO are human effect (29.2%), poor leadership (27.6%), and poor communication (23.1%) [16]. In fact, most risk factors for RFO/RSI are related to the quality of teamwork [10,17]. Poor teamwork and poor conflict resolution play a significant role in the creation of an unsafe operating room (OR) environment. In the OR, surgeons, anesthesiologists, and nurses disagree on their perception of teamwork. In a survey, OR nurses received the highest overall ratings for teamwork (4.20 of 5.00). Surgeons had the lowest overall ratings (3.68 of 5.00) followed by anesthesiologists (3.96 of 5.00) even though surgeons and anesthesiologists rated teamwork within their own disciplines the highest (4.38 of 5.00 and 4.80 of 5.00 respectively). OR nurses, however, rated teamwork with surgeons the lowest (3.52 of 5.00) [18,19]. There is also a disparity of opinion regarding the degree of success in conflict resolution. While conflicts are appropriately resolved according to 55% of anesthesiologists and 69% of surgeons, only 47% of the nurses feel that way [20]. Both these reports suggest a communication break between the different professions. Furthermore, the presence of a hierarchy and a lack of clarity of roles and responsibilities likely increase the risk of RFO incidents. This potential risk is further augmented if the surgeon is hurrying to leave the OR to get to the next surgery [21]. Fear of retribution likely plays a role as speaking up to report that the surgical count reveals a missing item can be very difficult, particularly for young, inexperienced, or temporary staff [22]. 

The very high percentage of wrong “correct counts” in the series reported by the Joint Commission is perplexing and confirms the critical role of the OR culture in the genesis of RFO [16]. A sponge count was performed in 77.4% (n=247/319) of all reported RFO incidents. When the count was performed, it was reported as being correct, when in fact, it was incorrect at 80.6% (n=199/247) of the time [15]. This high incidence of incorrect counts called “correct” may suggest that those counting could be scared that mentioning the count discrepancy would delay the procedure, and upset the surgeon and the anesthesiologist [21]. This latter explanation is more in line with the findings that 90% of RSO/RSI events are secondary to team/system failures and not individual failures [10]. Our own experience confirmed the role of contributing factors, such as unclear policies regarding the counting responsibility, the handling of surgical specimens, the involvement of two surgical teams, and the proper hand-off with the shift of the surgical technician. We observed an RFO occurring when the “confident” surgeon chose to override the written policy about sponge counting. Not surprisingly, in this ambiguous environment, team communication, particularly if involving new and inexperienced staff, becomes suboptimal [22]. 

We would argue that in a workplace like this, the RFO is a dead canary. Canaries were used to test for carbon monoxide in coal mines because these birds would show signs of distress even at relatively low CO levels. If the bird collapsed, the mine was to be evacuated before the situation became deadly. Just as the dead bird signaled the presence of lethal gas in the coal mine, the RFO signals the presence of a dangerous culture in the OR.

In order to prevent RFOs from occurring today, we can begin by ensuring standardization of what safety culture means to the entire organization [22]. Each level of the infrastructure present in healthcare (government and regulatory bodies, hospital, divisional management, OR staff, and work process) must share and embrace the key elements of safety culture [23]. Safety culture requires transparency and organization leaders who strengthen reporting near misses/close calls develop a stronger safety culture [24]. 

Complex non-medical industries have evolved incident reporting systems that focus on near misses, provide incentives for voluntary reporting, and ensure confidentiality while bolstering accountability. Reporting of near misses offers numerous benefits over the reporting of adverse events. Near misses do not cause harm to the patient. Due to the limited or no liability, there are much fewer barriers to collect data on them compared to sentinel events. Furthermore, near misses are much more frequent than adverse events and allow for quantitative analysis and detection of recovery patterns that can be captured, studied, and used for improvement [24-26]. Understanding what almost went wrong and identifying early signs of potential mistakes improve recovery strategies to avoid future occurrences [27].

Adoption of new technology, such as automatic counting using bar-coding surgical sponges, has been proven effective in reducing the retention of sponges [28,29]. Magnetic retrieval devices and sharp detectors are promising tools to improve the recovery of metallic RSI [25]. An integrative review synthesizing the evidence on the effectiveness of radiofrequency scanning reported its effectiveness in preventing RFOs with significant cost savings [30]. However, a systematic review examining what type of intervention has been implemented to prevent RFO and their effectiveness failed to corroborate the ability to reduce RFO, due to the heterogeneity in the intervention used and the variable study quality [31]. A study from the US reporting all RFOs out of a total of 997,237 procedures performed at five large urban hospitals from 2007 to 2017 determined that while the benefit of TeamSTEPPS (a crew resource management tool to improve teamwork) alone may not result in a reduction of RFOs, the incidence of RFOs was significantly lowered from 5.21 to 1.35 events per 100,000 operations (odds ratio [95% CI]=0.26 [0.11 to 0.60]) by the implementation of RF detection [32]. However, a study from Australia reported that due to a lack of data supporting efficacy and cost-effectiveness, adjunct technological RFO prevention strategies (using radiography, radiofrequency, and barcoding are not yet viable [33]. Implementing new technology alone, however, will not suffice to prevent RFO events without standardizing reports and improving team communication [25].

Once an RFO has occurred, the goal of the root cause analysis (RCA) process is to identify all contributing factors, initiate action plans to remove latent risk conditions, and successfully prevent reoccurrence. The development of an effective prevention strategy requires a systematic and in-depth RCA. It is paramount for the members of the RCA team to not simplify interpretations [34-36]. Simplifications are troublesome because they provide a false sense of understanding. When the RCA process simplifies the interpretation of a sentinel event (SE), it ends up recommending weak action plans (i.e. re-education or writing a policy) that are less likely to prevent recurrence of such events [36]. Performing a thorough RCA can be difficult or impossible because of existing barriers, such as lack of time, lack of resources, lack of data and feedback, uncooperative colleagues, difficulty with teams, interprofessional differences, and unsupportive management [35,36]. Performing a proper RCA requires commitment at all levels, starting from the very top of the organization.

Leadership must be committed, and act with vigor. “Commitment to resilience” means putting safety first, ensuring appropriate resources, and role modeling [34]. Lack of commitment to follow-up on the outcome of an RCA recommendation is a sign of poor leadership and constitutes another significant barrier in preventing the re-occurrence of these events [34-36]. 

In cases when an RCA discovers that a policy was ignored or violated, it behooves the team to ascertain if the OR policy itself was ambiguous. The OR work process could be made immediately safer by mandating the counting before the wound is closed, and not at the end of the procedure [21,22]. If a count is incorrect, it is both psychologically easier and operationally more efficient to re-explore while the wound is still open. “Deference to expertise” is key to creating strong policies that are understood and embraced by the frontline and that improve safety. This means inviting the OR staff to comment on the policy being discussed, encouraging equal attention to advice regardless of the seniority or “prestige” of the source, and valuing information quality rather than the attributes of the advice giver [37].

Maintaining safety requires reinforcing all the processes that produce collective mindfulness within the multidisciplinary team [38]. Activities aimed at strengthening resilience should become a habit [39]. Amateurs practice until they get it right, professionals practice until they cannot get it wrong. Every member of the team, namely the surgeon, the anesthesiologist, the nurses, the technicians, and the administrators should develop a healthy “preoccupation with failure” and personally feel accountable. Training should require that all the team members develop a “sensitivity to operations” seeing each other as indispensable elements of an integrated big picture [27,40]. This situational understanding can preempt the compounding of small problems by making timely corrective adjustments. Assessing group resilience, i.e., assessing how a team copes with everyday situations by adjusting performance under both expected and unexpected conditions should be a recurrent activity [39,40]. 

An unsafe OR culture is best addressed with a coordinated intervention predicated on aggressive coaching of the entire OR team. It is necessary to instill in the OR team the awareness that failure can and will happen [6]. It is critical to remind everyone of their individual responsibility in maintaining the safety of the complex system in which they work [38,40,41]. 

One of the ways that OR staff can be supported in their efforts is by creating a happy, respectful, and safe work environment to reduce the inevitable stress associated with work in the OR and allow their compassion to shine. Compassion benefits patients and their families by increasing engagement, compliance, and satisfaction. Compassion also lowers the risk of errors and protects staff from burnout by deepening relationships with colleagues, lowering stress, and making patient safety a team responsibility. Compassion includes recognition that mistakes hurt people and that those harmed deserve sincere apologies. Furthermore, when we make a mistake, a sincere apology will help us to prevent future harm.

Another area that should be reinforced during team coaching is the importance of being inquisitive and suspicious of simple explanations promoting a reluctance to simplify interpretations [6]. Coaching surgeons and anesthesiologists will support them in their responsibility to make the OR an environment that encourages everyone, regardless of their gender or position, to speak up when they have concerns [42-44]. There is often resistance among physicians, especially surgeons, to have their actions analyzed and judged in public forums. However, the confidential and safe space of intentional coaching allows participants to practice vulnerability without feeling the need to be defensive [42,44]. This exercise will help the surgeon to understand that, becoming the leader of the team means leading in safety. It is equally important to coach senior managers because their words and deeds receive enormous attention. They greatly influence how frontline workers, and middle managers perceive what the organization values and rewards [45]. In other words, to ensure long-term success, it is necessary to develop managers that people want to follow [46]. Coaching can engrain in minds that safety demands accountability, striking the right balance between blaming mistakes on systems and holding individual providers accountable. 

Everyone needs to actively participate in safety practices, such as hand-washing, preoperative antibiotics, protocols/checklists, reconciliation, time-out, reporting errors and adverse events (AEs), and identifying hazards and acting on them. Failure to follow clear rules is a violation and repeated violations in health care, as in any industry, have consequences. While it is unfair to blame individuals for team errors, there can be no tolerance for misconduct [47]. 

Similarly, it is necessary to remind leaders that it is unwise to prioritize productivity over safety. Productivity pressure and the strong economization of operating rooms create additional time pressure, fast-paced work processes, and high levels of stress in the OR. These are detrimental to creating the safety culture essential for RFO prevention [21]. Ensuring alignment of leadership with the culture of safety is mandatory. Leadership that strongly supports transparent reporting of adverse events (close calls and SEs), strengthening of the RCA process and action plans, heightened accountability, enhanced teamwork, and incorporation of available technology sustains an improved safety climate in the OR and in the organization [48-50]. Clearly, the repetition of the same adverse event suggests a manifest failure of the RCA process itself. 

## Conclusions

The journey towards the patients’ safety never ends and past performance cannot predict future safety. Healthcare organizations committed to safety assess and measure their safety culture and performance over time through employee surveys, by establishing and monitoring systems that track near-misses, errors, and adverse events, by conducting regular interviews and focus groups with staff and patients, by instituting regular "safety huddles" or "safety rounds", and by developing and tracking key performance indicators (KPIs) that measure specific aspects of safety and quality. Performing regular RCAs helps understand the underlying causes of significant adverse events. Failure mode and effect analysis (FMEA) offers a proactive tool to identify potential points of failure in a system before they lead to an adverse event. Other safety assessments require benchmarking, external audits, and patient and family feedback. Lastly, simulation and training are invaluable tools to enhance safety.

Safety is everyone’s responsibility, and it is preserved by timely human intervention. Despite the implementation of RFID labeling, magnetic retrieval devices, and sharp detectors, RFOs continue to occur because technology alone is insufficient and will not succeed without improving team communication and collaboration. Every member of the team, namely the surgeon, anesthesiologists, nurses, technicians, and administrators needs to feel accountable. Ongoing interaction and information sharing increase the safety of the system. OR staff that are key participants in the review of policies help make policies relevant and clear. When an RFO occurs, teams that question assumptions create a clearer picture of what happened. Commitment to resilience means putting safety first by creating a respectful environment, boosting collaboration, and keeping productivity pressure under check, thus preventing unnecessary stress detrimental to the creation of safety in the OR. An organization can become safe when the people working in it are certain that their patients are as safe as humanly possible in the organization, and that all personnel are collaborating to make healthcare safe.
